# Comparative Analysis of Biological Characteristics among P0 Proteins from Different Brassica Yellows Virus Genotypes

**DOI:** 10.3390/biology10111076

**Published:** 2021-10-21

**Authors:** Xiao-Yan Zhang, Yuan-Yuan Li, Ying Wang, Da-Wei Li, Jia-Lin Yu, Cheng-Gui Han

**Affiliations:** 1 School of Agriculture, Ludong University, Yantai 264025, China; xiaoyan433@163.com; 2 State Key Laboratory for Agrobiotechnology, Ministry of Agriculture and Rural Affairs Key Laboratory of Pest Monitoring and Green Management, China Agricultural University, Beijing 100193, China; ynyli@ucdavis.edu (Y.-Y.L.); yingwang@cau.edu.cn (Y.W.); Lidw@cau.edu.cn (D.-W.L.); yjl@cau.edu.cn (J.-L.Y.); 3 Department of Plant Biology and The Genome Center, College of Biological Sciences, University of California, Davis, CA 95616, USA

**Keywords:** Brassica yellows virus, P0, RNA silencing suppressor, cell death

## Abstract

**Simple Summary:**

Polerovirus P0 proteins are multifunctional proteins. Besides their viral suppressor of RNA silencing (VSR) functions, several P0 proteins can induce a cell death phenotype within the infiltrated region of *Nicotiana benthamiana* or *Nicotiana* *glutinosa*. Recently, the Brassica yellows virus (BrYV) genotype A P0 protein (P0^BrA^) was identified as a strong viral suppressor of RNAi. In this study, we compared the features of the P0 proteins encoded by different genotypes of BrYV and revealed their difference in inducing cell death in *N. benthamiana*. Key residues in P0^BrA^ for inducing cell death were also identified. We also showed that all three BrYV genotypes had synergistic interaction with PEMV 2 in *N. benthamiana*. This study provides theoretical guidance for controlling the viral disease caused by poleroviruses in the future.

**Abstract:**

Brassica yellows virus (BrYV) is a tentative species of the genus *Polerovirus*, which has at least three genotypes (A, B, and C) in China. The P0 protein of BrYV-A (P0^BrA^) has been identified as a viral suppressor of RNA silencing (VSR), which can also induce cell death in infiltrated *Nicotiana benthamiana* leaves. In this study, we demonstrated that the cell death induced by P0^BrA^ was accompanied by the accumulation of reactive oxygen species (ROS) and increased *Pathogenesis-related protein genes-1* (*PR1*) expression. Meanwhile, this cell death phenotype was delayed by salicylic acid (SA) pretreatment. Biological function comparison of the three P0 proteins showed that transiently expressed P0^BrB^ or P0^BrC^ induced a significantly delayed and milder cell death response compared with P0^BrA^. However, like P0^BrA^, they also suppressed local and systemic RNA silencing. Six residues of P0^BrA^ essential for inducing cell death were identified by comparative analysis and amino acid substitution assay. We also show that all three BrYV genotypes have synergistic interactions with pea enation mosaic virus 2 (PEMV 2) in *N. benthamiana*. This study provides theoretical guidance for controlling the viral disease caused by poleroviruses in the future.

## 1. Introduction

RNA silencing is an important immune strategy used by plants against plant viruses [1]. To suppress the host anti-viral RNA silencing, many plant viruses have evolved viral suppressor of RNA silencing (VSR) proteins [2,3,4,5]. The P0 proteins have been identified as VSR in many poleroviruses, including *Turnip yellows virus* (TuYV), *Cucurbit aphid-borne yellows virus* (CABYV), *Potato leafroll virus* (PLRV), *Beet mild yellowing virus* (BMYV), *Cotton leafroll dwarf virus* (CLRDV), *Sugarcane yellow leaf virus* (ScYLV), *Melon aphid-borne yellows virus* (MABYV), *Maize yellow mosaic virus* (MaYMV), *Cereal yellow dwarf virus* (CYDV), *Wheat yellow dwarf virus*-GPV isolate (WYDV-GPV), Maize yellow dwarf virus-RMV 2 (MYDV-RMV2), and Pea mild chlorosis virus (PMCV) [6,7,8,9,10,11,12,13,14,15,16,17,18,19,20,21]. The amino acid sequence of P0 proteins throughout the genus shows low identity and the RNA silencing suppressor (RSS) activity of the P0 proteins varies between different viruses. Among the P0 proteins mentioned above, those encoded by PLRV, ScYLV, CYDV, MaYMV, and MYDV-RMV2 display systemic silencing suppression activity, while P0 proteins of TuYV, CABYV, and CLRDV do not have systemic silencing suppression activity [7,8,14,17]. Different isolates of the same virus (BMYV and PLRV) can exhibit various efficiencies in RSS activity [11,14,22]. P0 proteins of several polerovirus members can trigger ARGONAUTE1 degradation via the autophagy pathway to suppress RNA silencing [6,16,23,24]. Studies have shown that P0 proteins of TuYV, CABYV, CYDV-RPV, CYDV-RPS, and PMCV have a consensus F-box-like domain, through which P0 proteins interact with S-phase kinase-associated protein 1 (SKP1), a component of the SKP1–Cullin F box (SCF) family of the E3 ubiquitin ligases complex [10,18]. Although PLRV and PMCV P0 proteins contain a conserved F-box-like domain and suppress RNA silencing, they fail to interact with SKP1 [6,11].

Several polerovirus P0s are multifunctional proteins. Besides their VSR functions, the P0 proteins of TuYV (P0^Tu^), CABYV (P0^CA^), PLRV (P0^PL^), ScYLV (P0^Sc^), and CYDV (P0^CY^) are reported to induce the cell death phenotype within the infiltration region in *Nicotiana* species [14,16,17,25]. The cell death phenotype induced by P0 proteins varies among different viruses. Under the same experimental conditions, the P0^CY^ protein triggered an obvious cell death phenotype of about 7 dpi; however, P0^PL^ triggered necrosis that began developing around 14 dpi [14]. A previous study by Mangwende et al. demonstrated that P0^Sc^ triggered the cell death phenotype as early as 1 dpi. P0^Sc^ and P0^Tu^ induced a dosage-dependent cell death phenotype in infiltrated *N. benthamiana* plants while this phenotype was not observed in P0^PL^ or P0^CA^ [16,17]. Meanwhile, P0^Tu^, P0^PL^, and P0^CA^ were reported to elicit hypersensitive responses (HR) in *Nicotiana glutinosa*, and genetic analysis revealed the recognition of P0^Tu^ by the *RPO1* resistance gene [25].

Brassica yellows virus (BrYV) is a tentative species of the genus *Polerovirus*, which is widespread throughout China, South Korea, and Japan [26,27,28,29]. Its positive-sense single-stranded RNA (+ssRNA) genome is approximately 5.7 kb in size and includes seven open reading frames (ORFs) encoding seven proteins [30]. The BrYV has at least three different genotypes (A, B, and C) and the infectious cDNA clones of the three BrYVs have been successfully developed [31,32]. A large-scale survey of the incidence and prevalence of BrYV in China showed that the three BrYV genotypes displayed differences in incidence rates and host species [26]. Recently, the BrYV genotype A P0 protein (P0^BrA^) was identified as a strong viral suppressor, exhibiting both local and systemic RSS activity. The P0^BrA^ can interact with SKP1, which is beneficial for P0^BrA^ stabilization. This ensures efficient RSS activity that is required for the BrYV effective systemic infection of *N. benthamiana* plants [30]. Moreover, P0^BrA^ can induce cell death within the region of infiltration [30]. According to previous sequence comparisons, the 5′-proximal ORF0 of the three genotypes shares 90.4–92.5% nucleotide sequence identity and 86.7–90.8% amino acid sequence identity [32]. Therefore, there may be some differences in their biological functions, but whether these biological functions are related to differences in incidence levels of the three BrYV genotypes is not known. This study aimed to investigate whether P0 of the three BrYV genotypes displayed similar biological functions. We assessed and compared the local and systemic RNA silencing suppression activities of different BrYV genotypes. In addition, several residues in P0^BrA^ essential for inducing cell death were also identified through P0 protein comparative analysis.

## 2. Materials and Methods

### 2.1. Plant Material and Growth Conditions

Wild-type *N. benthamiana* and green fluorescent protein (GFP) transgenic *N. benthamiana* line 16c were grown in a climate chamber with a 16-h photoperiod at 24 °C.

### 2.2. Plasmid Constructs

The primers used in this study are shown in Supplementary Table S1. The pGD, pGDG, and pGD-P0^BrA^-3Flag vectors were used for transient expression [30,33]. P0^BrB^, P0^BrC^, and P0^BrA^ mutants were amplified by PCR and cloned into to pGD-3Flag vector, which fused with a C-terminal 3 × Flag tag to produce the pGD-P0^BrB^-3Flag, pGD-P0^BrC^-3Flag, and desired P0^BrA^ mutants [34], respectively. For the PVX constructs, heterologously expressing P0^BrA^ was amplified with primers BrAP0ClaF/BrAP0SalhisR and then cloned between the *Sal*I and *Cla*I sites of the pND108 vector [35]. Full-length cDNA infectious clones of BrYV (pCaBrA, pCaBr5B3A, and pCaBrC) and PEMV 2 (pCaPE2) were constructed as described [31,36].

### 2.3. Transient Co-Expression Assay and GFP Fluorescence Observation

The constructs were transformed into the C58CI strain of *Agrobacterium tumefaciens* using the freeze–thaw method [33]. Co-infiltration assays were performed as described previously [37,38]. *A. tumefaciens* cultures containing pGDG expressing GFP and *A. tumefaciens* cultures containing pGD-P0^BrB^-3Flag, pGD-P0^BrC^-3Flag, or pGD-P0^BrA^-3Flag were mixed and co-infiltrated into *N. benthamiana* leaves. For the mutagenesis experiments, *A. tumefaciens* cultures harboring the relevant binary plasmids were mixed prior to infiltration. The concentration for mixed infiltrations at OD_600_ was 0.5 for each. GFP fluorescence was measured under a long-wavelength UV lamp and images were recorded with a digital camera under a yellow filter at 2 and 14 dpi, respectively.

### 2.4. Agrobacterium-Mediated Inoculation of Virus

The pCaBrA, pCaBr5B3A, pCaBrC, or pCaPEMV 2 plasmids were transformed into *A. tumefaciens* strain GV3101 and infiltrated into 4-week-old *N. benthamiana* plants. For single virus infiltration, the concentration of cell suspension was 0.5 at OD_600_. For mixed infiltrations, the OD_600_ was 0.5 for each.

For PVX and PVX.P0^BrA^ inoculation, the plasmids were transformed into the *A. tumefaciens* strain GV3101 and infiltrated into 4-week-old *N. benthamiana* plants. The infiltration was at a concentration of 0.1 at OD_600._ Upper leaves were harvested at 6 dpi and analyzed. PVX.P0^BrA^ was a recombinant PVX-based vector expressing the P0^BrA^ protein. The empty PVX vector was used as the control.

### 2.5. Western Blot Analysis

Total protein extraction and Western blotting were performed as described [11]. Protein samples were separated by electrophoresis in 12.5% SDS-PAGE and transferred onto a Nitrocellulose Membrane (GE Healthcare, Chicago, IL, USA). The antiserum against GFP was used to detect GFP expression, and the Flag antibody (Sigma-Aldrich) was used to detect expression of the P0^BrA^, P0^BrB^, P0^BrC^, or P0^BrA^ mutants. After incubation with primary antibody and washing, the membrane was incubated with goat anti-rabbit alkaline phosphatase-conjugated secondary antibody (Sigma-Aldrich, St. Louis, MO, USA) followed by NBT (0.33 mg/mL)/BCIP (0.165 mg/mL) staining.

### 2.6. Diaminobenzidine (DAB) Staining

Agroinfiltrated leaves of *N. benthamiana* were harvested and incubated in 1 mg/mL DAB in the dark for 8 h and destained with 96% ethanol.

### 2.7. Reverse Transcription PCR and Northern Blotting

Plant total RNA was prepared by an SDS-phenol/chloroform extraction and a reverse transcription (RT) reaction was conducted as previously described [26,30]. BrYVs and PEMV 2 were detected using the primers BrYA484F/BrYB88F/BrYC257F/BrY761R and PEM2797F/PEM3202R, respectively. After PCR amplification, the products were separated in 1.5% agarose gels and stained with ethidium bromide.

Northern blot was performed as described [36]. RNAs used for BrYV (3 μg) and PEMV 2 (5 μg) were separated in a 1.2% formaldehyde-agarose gel and then transferred onto a Hybond-N^+^ nylon membrane. Prehybridization was performed for 5 h at 65 °C. The [α-^32^P] dCTP-labeled DNA probe specific for BrYV or PEMV 2 was generated using the Prime-a-Gene labeling system (Promega, Madison, WI, USA). Hybridization was carried out at 65 °C for 16 h. After washing, the nylon membrane was exposed to a storage phosphor screen (GE healthcare).

### 2.8. Real-Time Quantitative PCR

Total RNA was extracted using Trizol Reagent (Invitrogen, Waltham, MA, USA) according to the manufacturer’s protocol. cDNA was synthesized from 3 μg total RNA using an oligo(dT) primer and M-MLV reverse transcriptase (Promega). The gene fragments were amplified using 2 × SsoFastTM EvaGreen Supermix (Bio-Rad, Hercules, CA, USA). Primers 212F22/341R23 shown in Table S1 were used for amplification of the *NbPR1* gene [39]. 18S ribosomal RNA gene served as an internal control by using the primers 18S-1/18S-2 [39]. *NbPR1* gene expression was normalized to the 18S ribosomal RNA gene and the data were analyzed using CFX MANGE software (Bio-Rad).

### 2.9. Salicylic Acid (SA) Pre-Treatment Assay

Five-week-old *N. benthamiana* plants were sprayed and soil drenched with a solution of 1.1 mM SA in 0.11% ethanol 4 days prior to infiltration. On Day 5, *Agrobacterium* harboring the construct PVX.P0^BrA^ or an empty vector was infiltrated into the treated leaves and sprayed with SA again 4 h later. Samples were collected 2 days after SA treatment. Control plants were sprayed with 0.11% ethanol.

## 3. Results

### 3.1. Cell Death Induced by P0^BrA^ Was Accompanied by Increased Production of ROS and Induction of PR1 Expression

**Figure 1:**
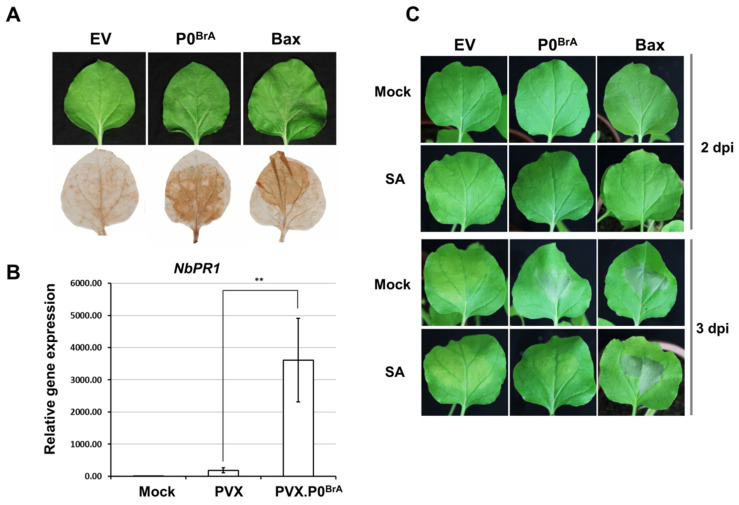
Transient overexpression of P0^BrA^-triggered HR-like cell death in *N. benthamiana* leaves. (**A**) DAB staining in *N. benthamiana* leaves transiently expressing P0^BrA^ at 48 h post infiltration. Empty vector (EV), negative control; Bc1-2-associated X protein (Bax), positive control. (**B**) Analysis of *PR1* transcript accumulation in *N. benthamiana* plants infected with PVX or PVX.P0^BrA^ by qRT-PCR. Values are presented as the mean ± standard error (*n* = 3). ** *p* < 0.01; asterisks show significant differences as determined using the Student’s *t*-test. Error bars represent standard errors of the means. (**C**) Effect of SA treatment on cell death phenotypes triggered by P0^BrA^. EV, negative control. Bax, positive control. A 0.11% ethanol treatment (Mock) was used as the solvent control.

To investigate whether the cell death caused by P0^BrA^ is a plant immune response, we analyzed the markers of plant immunity. DAB staining detected the accumulation of ROS, a physiological response associated with the onset of HR, in the infiltration regions of *N. benthamiana* leaves expressing the P0^BrA^ protein (Figure 1A). Similar to the Bc1-2-associated X protein (Bax, positive control), the expression of which triggered cell death phenotype and ROS accumulation [40], transient expression of P0^BrA^ protein was also followed by ROS accumulation. The phytohormone salicylic acid (SA) plays an important role in regulating plant immunity and its accumulation induced the expression of a series of downstream genes, including *Pathogenesis-related protein genes-1* (*PR1*). In the systemic leaves of *N. benthamiana* plants infected with PVX.P0^BrA^ (a recombinant PVX-based vector expressing the P0^BrA^ protein), the *PR1* gene transcription level was significantly increased compared with the control plants (Figure 1B). Interestingly, we observed a significantly delayed cell death response in the *N. benthamiana* plants with exogenous SA pretreatment compared with the control plant pretreated with ethanol (Figure 1C). These results suggest that the cell death induced by P0^BrA^ in *N. benthamiana* may be the plant immune response to the virus.

### 3.2. Both P0^BrB^ and P0^BrC^ Suppressed Local and Systemic RNA Silencing

**Figure 2:**
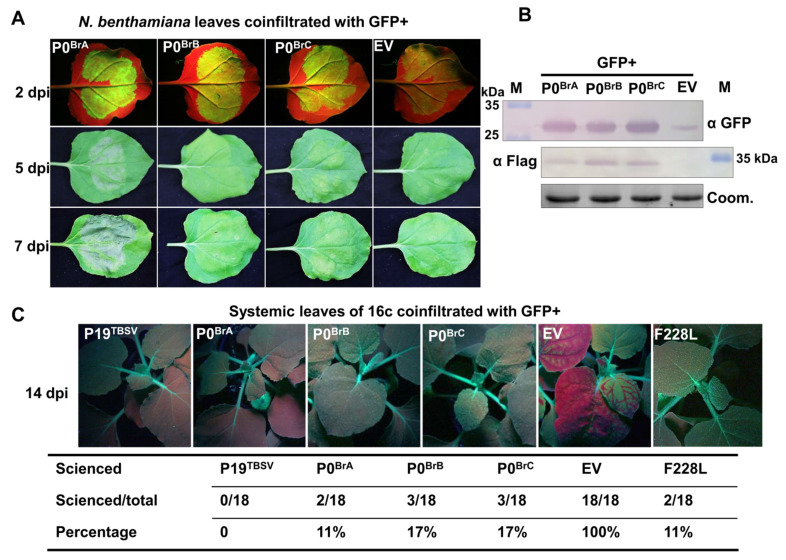
Suppression of local and systemic RNA silencing by P0^BrB^ and P0^BrC^. (**A**) Suppression of local GFP silencing in *N. benthamiana*-infiltrated leaves. Agroinfiltration with GFP and an empty vector pGD (EV), P0^BrA^, P0^BrB^, or P0^BrC^. The leaves infiltrated with EV were included as the negative control. (**B**) Western blot analysis of GFP and three Flag-tagged P0^Br^ protein levels in infiltrated *N. benthamiana* leaf patches using an antibody specific for GFP and Flag, respectively. Coomassie-stained (Coom.) gels are the loading control. M, molecular weights of protein marker. (**C**) Systemic RNA silencing suppression activity of P0^BrB^ and P0^BrC^. Transiently coexpressed GFP and P0 were in leaves of *N. benthamiana* line 16c. P19^TBSV^ and EV were used as positive and negative controls, respectively. The efficiency of systemic silencing is presented below the images. The ratio shows the number of systemic silencing plants to the number of infiltrated plants, scored in three independent experiments.

BrYV was identified to have at least three genotypes in China [26,31]. We previously identified BrYV-A P0 as a strong viral suppressor of RNA silencing, with both local and systemic RNA silencing suppression activity [30]. To confirm whether P0 encoded by the BrYV genotypes B and C (P0^BrB^ and P0^BrC^) are also RNA silencing suppressors, P0^BrA^, P0^BrB^, or P0^BrC^ were transiently coexpressed together with a green fluorescent protein (GFP) in *N. benthamiana* leaves [38]. Under the long-wavelength UV light at 2 days post-infiltration (dpi), the leaf patches co-infiltrated with GFP and the empty vector (EV) showed faint GFP fluorescence, suggesting that GFP RNA silencing was effectively induced (Figure 2A). In contrast, the GFP/P0^BrB^ or GFP/P0^BrC^ co-infiltrated leaf patches showed strong GFP fluorescence, similar to GFP/P0^BrA^ (Figure 2A). The accumulation of the GFP protein was detected using Western blot. In GFP/EV-infiltrated leaf patches, the GFP proteins were rarely detected. Furthermore, a high accumulation of GFP was detected in GFP/P0^BrB^- and GFP/P0^BrC^-infiltrated leaf patches, similar to the GFP/P0^BrA^ (Figure 2B). These results suggest that P0^BrB^ and P0^BrC^ suppressed local RNA silencing in *N. benthamiana*.

To test whether P0^BrB^ and P0^BrC^ could prevent the spread of the systemic silencing signal, a GFP co-infiltration assay was conducted in transgenic *N. benthamiana* line 16c. In this experiment, GFP transient expression in the lower leaves resulted in GFP silencing in the newly emerging leaves, which could be monitored by UV illumination [41]. At 14 dpi, the fluorescence signals of GFP in the upper leaves were observed under the long-wavelength UV light (Figure 2C). By calculating the percentage of systemic silencing suppression, the result showed that all the plants co-infiltrated with GFP/EV (negative control) exhibited systemic RNA silencing. Conversely, no plants co-infiltrated with GFP and P19^TBSV^, which served as the positive control, exhibited systemic RNA silencing at 14 dpi [42]. Only 11, 17, and 17% of the plants infiltrated with GFP/P0^BrA^, GFP/P0^BrB^, and GFP/P0^BrC^ exhibited systemic RNA silencing, respectively (Figure 2C). These data showed that P0^BrB^ and P0^BrC^ display a systemic RNA silencing suppressor activity similar to P0^BrA^.

### 3.3. P0^BrB^ and P0^BrC^ Induce Significantly Delayed and Milder Cell Death in N. benthamiana Compared to P0^BrA^

It was previously shown that P0^BrA^ induces cell death in infiltrated *N. benthamiana* leaves [30]. To confirm whether P0^BrB^ or P0^BrC^ also induce cell death, we transiently expressed them in *N. benthamiana* plants. At 5 dpi, the P0^BrA^ protein triggered an obvious cell death phenotype and rapidly developed severe necrosis by 7 dpi (Figure 2A). However, under the same conditions, the cell death phenotype was not evident in leaves expressing P0^BrB^ or P0^BrC^ at 5 dpi. Even at 7 dpi, both P0^BrB^ and P0^BrC^ only triggered a mild cell death response, which was significantly weaker than the P0^BrA^-induced cell death (Figure 2A). Immunoblotting results showed that the accumulation level of P0^BrB^ or P0^BrC^ was equivalent to P0^BrA^, indicating that dosage was not the reason for the compromised cell death (Figure 2B).

### 3.4. Identification of Key Amino Acid Residues in P0^BrA^ That Affect the Induction of Cell Death in N. benthamiana

**Figure 3:**
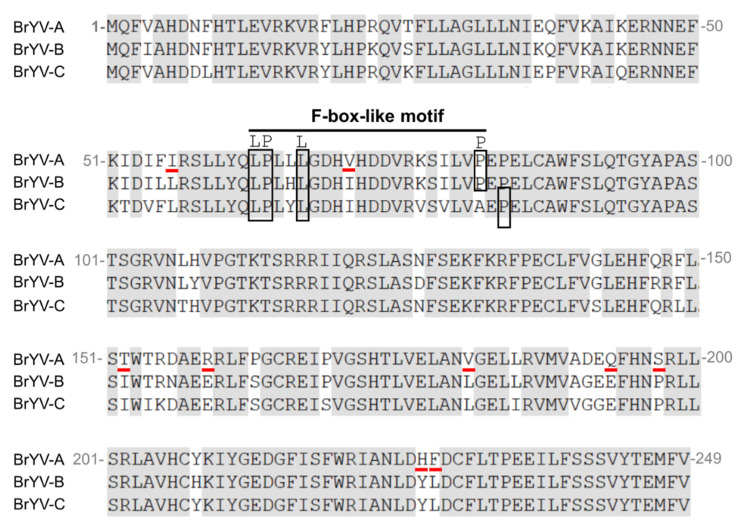
Multiple alignments of amino acid sequences from P0^BrA^, P0^BrB^, and P0^BrC^**.** All the conserved residues are indicated in gray background. The mutated amino acids of the nine mutants are presented by red underlines. Landmark residues of the F-box-like motif are highlight in the boxes. The P0 sequences used are as follows: BrYV-A (Accession No. HQ388348), BrYV-B (Accession No. HQ388351), and BrYV-C (Accession No. KF015269). The numbers correspond to the positions of amino acids within the P0^Br^ sequence.

**Figure 4:**
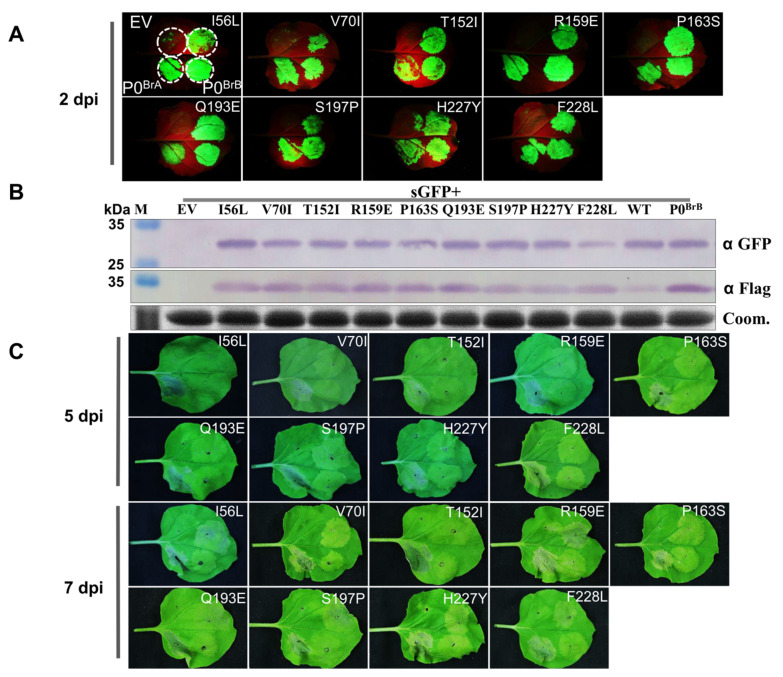
Suppression of local RNA silencing by P0^BrA^ mutants. (**A**) Agroinfiltration with GFP plus empty vector pGD (EV), P0^BrA^, P0^BrA^ mutants, or P0^BrB^ in *N. benthamiana* leaves. (**B**) Western blot analysis of GFP and P0^BrA^ mutants in *N. benthamiana*-infiltrated patches from (**A**). (**B**) GFP and P0^BrA^ mutants were detected using an antibody specific for GFP and Flag, respectively. Coomassie-stained (Coom.) gels are the loading control. Molecular weights of protein marker (M) are shown on the left. (**C**) Cell death phenotypes in *N. benthamiana* leaves with transiently-expressed P0^BrA^ or its mutants. Photographs were taken under white light to show cell death at 5 or 7 dpi. Empty vector (EV) was used as the negative control.

Sequence comparison of P0^BrA^, P0^BrB^, and P0^BrC^ showed that the shared amino acid sequence identity ranged from 86.7 to 90.8%. The amino acid sequence of P0^BrB^ and P0^BrC^ had nine residues, different from P0^BrA^ (Figure 3). In order to identify the P0^BrA^ key amino acids that function in inducing cell death, a single amino acid substitution in P0^BrA^, mutagenesis experiments were conducted for the following nine residues: Leu substitution of Ile56 (I56L), Ile substitution of Val70 (V70I), Ile substitution of Thr152 (T152I), Glu substitution of Arg159 (R159E), Ser substitution of Pro163 (P163S), Glu substitution of Gln193 (Q193E), Pro substitution of Ser197 (S197P), Tyr substitution of His227 (H227Y), and Leu substitution of Phe 228 (F228L) (Figure 3). The P0^BrA^ mutants were transiently coexpressed together with GFP in wild-type *N. benthamiana* leaves. Under the long-wavelength UV light, we found that the GFP fluorescence in the leaf patches co-infiltrated with pGDG and all nine mutants were as strong as the co-infiltration with pGDG and the wild-type P0^BrA^ at 2 dpi (Figure 4A). The GFP protein accumulation (Figure 4B) detected by Western blot corresponded to the GFP fluorescence levels (Figure 4A), indicating the nine substitutions in P0^BrA^ did not affect the local RNA silencing suppressor activity.

We then compared the cell death induction capacity of the nine mutants with P0^BrA^. At 5 dpi, the leaves co-infiltrated with P0^BrA^ showed a more prominent cell death phenotype than leaves infiltrated with mutants (Figure 4C). At 7 dpi, cell death was observed in parts of leaves expressing mutants I56L, R159E, and S197P (Figure 4C), indicating that the three mutations delayed the onset of rapid and strong cell death induced by P0^BrA^. The cell death phenotypes were not evident in infiltrations with the remaining six mutations V70I, T152I, P163S, Q193E, H227Y, and F228L (Figure 4C), suggesting that the Val70, Thr152, Pro163, Gln193, His227, and Phe 228 of P0^BrA^ were key residues essential for inducing cell death in *N. benthamiana* but not for suppressing local RNA silencing.

### 3.5. All Three BrYV Genotypes Have Synergistic Interaction with PEMV 2 Resulting in Increased Accumulation of BrYV and Causing Severe Symptoms in N. benthamiana

**Figure 5:**
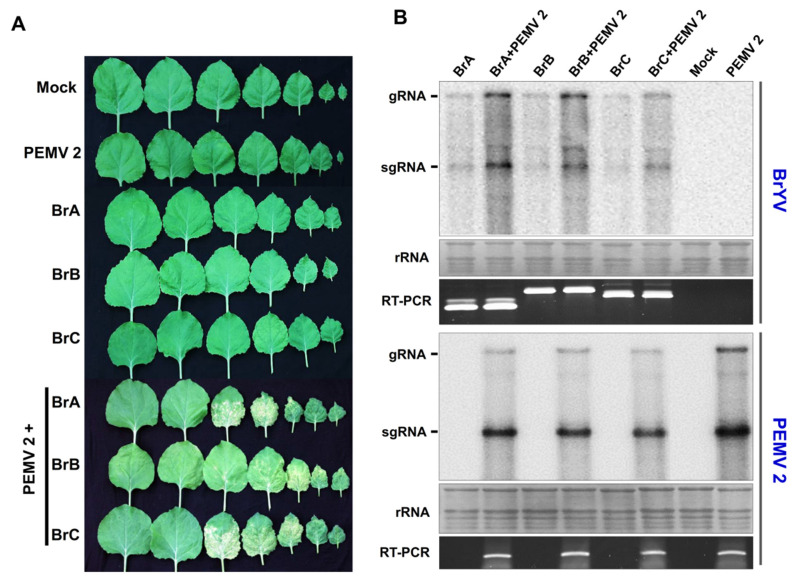
Systemic infection of the three BrYV genotypes and PEMV 2. (**A**) The symptoms of *N. benthamiana* upper leaves were induced by coinfection with BrYVs and PEMV 2. The upper leaves were photographed at 21 dpi. (**B**) Accumulation of BrYV and PEMV 2 in *N. benthamiana* upper leaves was analyzed using Northern blotting. The viral genomic RNAs (gRNA) and subgenomic RNAs (sgRNA) are shown on the left of the panel. Methylene blue-stained ribosomal RNA (rRNA) was used as the loading control.

As the BrYV is restricted to the host phloem tissue, the *Pea enation mosaic virus 2* (PEMV 2), which belongs to *Umbravirus*, can help BrYV move out of the phloem into nonvascular tissues and be transmitted mechanically [36]. Meanwhile, the synergistic infection of BrYV and PEMV 2 on *N. benthamiana* upper leaves produced severe symptoms and significantly increased the BrYV titer [36]. To further investigate whether the three BrYV genotypes display differences in synergism with PEMV 2, the wild-type plants were infiltrated with BrA, BrA + PEMV 2, BrB, BrB + PEMV 2, BrC, BrC + PEMV 2, empty vector (Mock), or PEMV 2. At 21 dpi, *N. benthamiana* upper leaves infected with BrA + PEMV 2, BrB + PEMV 2, and BrC + PEMV 2 all displayed necrosis and leaf-curling symptoms. In contrast, no obvious symptoms were observed in the plants infected with BrA, BrB, BrC, or PEMV 2 (Figure 5A). Northern blot analysis showed an increased accumulation of BrYVs (BrA, BrB, or BrC) in coinfected plants in contrast to BrYV-only infected plants (Figure 5B). In this experiment, sequence progeny for BrA, BrB, or BrC in the upper leaves was determined by multiplex RT-PCR followed by amplicon sequencing. The PEMV 2 infection was confirmed by RT-PCR and Northern blot detection (Figure 5B). Taken together, PEMV 2 displayed a synergistic interaction with all of the three BrYV genotypes, resulting in increased accumulation of BrYV and causing severe symptoms in *N. benthamiana*.

## 4. Discussion

P0 proteins have been previously identified as viral suppressors of RNA silencing in many poleroviruses. Recently, we showed that BrYV-A P0 is a strong VSR that can suppress both local and systemic RNA silencing [30]. Besides the VSR functions, the P0 proteins of TuYV, ScYLV, CABYV, and PLRV were reported to cause cell death phenotypes in some *Nicotiana* species. P0^Tu^, in particular, elicited the HR in *N. glutinosa*, which is recognized by the *RPO1* resistance gene, a likely immune receptor of P0 [16,17,25]. HR is a form of programmed cell death that accompanies defense reactions such as the generation of ROS, including H_2_O_2_, the expression of pathogenesis-related (PR) genes, and the accumulation of phytohormones, including salicylic acid (SA), jasmonic acid, and ethylene [43,44]. In this study, we discovered that transiently expressed P0^BrA^ in *N. benthamiana* leaves induced a cell death response. This rapid and restricted cell death phenotype is accompanied by the accumulation of ROS and the induction of the marker gene *PR1* expression, which is similar to the HR-like cell death in plants. The phytohormone SA plays an important role in the regulation of plant immunity [45]. Our study showed the P0^BrA^-induced cell death phenotype could be delayed by SA pretreatment, indicating that the SA pathway may be involved in cell death initiation, but we cannot rule out the involvement of other defense pathways. Generally, the development of HR resistance is associated with the recognition of the viral protein by the corresponding resistance (R) proteins of plants in a host-specific manner [46]. The rapid cell death induced by P0^BrA^ in the agroinfiltrated region of *N. benthamiana* leaves may be the result of P0^BrA^ recognition by an R protein from *Nicotiana* species, but further experiments are required to confirm this.

BrYV has a widespread distribution and prevalence in crucifer crops throughout China. According to the multiplex RT-PCR amplification, the three BrYV genotypes had differences in incidence rates and host species [26]. Among all the BrYV infection types, genotype C was detected at the greatest proportion, followed by genotype B and A. BrYV-C had the greatest incidence rates in five *Brassica* species: *B. rapa* var. *pekinensis*, *B. oleracea* var. *capitata*, *B. oleracea* var. *botrytis*, *B. oleracea* var. *alboglbra*, and *B. napus* [26]. *Raphanus sativus* and *B. juncea* plants have been previously infected with BrYV-A and BrYV-B genotypes [26]. As the three genotype sequences of BrYV ORF0 have a high diversity, a comparative analysis of the biological activities of their respective P0 proteins was done. We found that the P0 proteins of the two other BrYV genotypes (P0^BrB^ and P0^BrC^) also suppressed both local and systemic RNA silencing, such as P0^BrA^. However, transiently-expressed P0^BrB^ or P0^BrC^ induced a significantly delayed and milder cell death response compared with P0^BrA^. As the VSR of BrYV, P0 can suppress the host anti-viral RNA silencing and benefit the accumulation of virus. However, this pro-viral factor may possibly be recognized by plant hosts as an effector and elicit HR-like cell death response to restrict the spread of virus. Although P0^BrA^, P0^BrB^, and P0^BrC^ have similar VSR activities, P0^BrB^ or P0^BrC^ induced a significantly delayed and milder cell death response compared with P0^BrA^. This may imply the escape of BrYV genotypes C and B from the perception by host innate immune receptors, which may further facilitate their infection in plants. Consistent with this presumption, our previous study had shown that the BrYV genotype C and B have a higher incidence in nature compared with genotype A, which indicated that BrYV-C and B gradually become the dominant genotypes of BrYV during virus evolution. Moreover, it cannot rule out the possibility that the aphids are more likely to feed on the plants infected with BrYV-C or B in fields, which further facilitate the virus transmission. However, whether the aphid viral acquisition rates are related to P0’s cell death induction function needs further study.

Previous mutational analyses of P0^BrA^ revealed that the LP63-64AA mutant abolished local RNA silencing suppression (RSS) activity but retained the systemic RSS and cell death induction activity. In contrast, the L184A mutant retained local RSS activity but eliminated the systemic RSS and cell death induction activity, suggesting that the cell death induction of P0 is independent of the local suppression activity [30]. In this study, the six mutations V70I, T152I, P163S, Q193E, H227Y, and F228L suppressed local PTGS but failed to induce the obvious cell death phenotype. The mutant F228L in particular also suppressed systemic PTGS (Figure 2C), suggesting that the cell death induction activity was not directly required for P0 to suppress systemic RNA silencing. Since the Phe228 plays an important role in cell death induction, the screening of host proteins interacting with the F228L mutant, but not with the wild-type protein, may provide clues for the underlying mechanism of P0^BrA^ in inducing cell death. However, further research is also needed to determine whether the F228L mutation affected the viral infection.

Earlier studies have shown that the P0 proteins in poleroviruses have a conserved F-box-like motif [LP_XX_(L/I)_X10–13_P] and FWR motif [(K/R) IYGEDGX_3_FWR], indispensable to the silencing suppression activity [6,8,9,10,11,12,14,15,17,18,24,30]. Multiple alignments of the P0 proteins from three BrYV genotypes showed that they shared an amino acid sequence identity, ranging from 86.7 to 90.8% [31]. A highly conserved FWR motif (209-KIYGEDGFISFWRIA-223) was present in the three P0^Br^ proteins (Figure 2). P0^BrA^ and P0^BrB^ had a similar F-box-like motif (63-LPL(L/H)LGDH(V/I)HDDVRKSILVP-82). Interestingly, the amino acid residue 82 of P0^BrC^ in the F-box-like motif was an alanine (A) instead of the consensus proline (P), but residue 84 of P0^BrC^ was a P (Figure 2). We speculate that 84P is the key site for the F-box-like motif of P0^BrC^, but further investigation is required. Our previous studies have shown that P0^BrA^ interacts with SKP1 via its F-box-like motif to stabilize itself, ensuring efficient RSS activity of P0^BrA^ for BrYV infection [30]. Studies have shown that both P0^PL^ and P0^PM^ have conserved F-box-like motif and suppress RNA silencing but fail to interact with SKP1. Therefore, it will be interesting to investigate the difference in SKP1-interacting abilities among P0^BrA^, P0^BrB^, and P0^BrC^.

Viral synergism is common in nature [47,48,49,50,51,52,53,54]. The synergistic infection of phloem-restricted poleroviruses and umbraviruses has destructive effects on crop plants. Previous research has shown that in the presence of PEMV 2, BrYV can move out of the phloem to invade the mesophyll tissues and be transmitted mechanically in *N. benthamiana* [36]. Our results presented that the three BrYVs have no obvious difference in synergism with PEMV 2. All three BrYV genotypes have synergistic interactions with PEMV 2, resulting in increased accumulation of BrYV and thus causing more severe symptoms in *N. benthamiana*. As an RNA silencing suppressor, P0 is needed for BrYV systemic infection [30]. However, whether the P0 protein is necessary for synergism between BrYV and PEMV 2 is uncertain. Further investigation is also needed to identify if the P0 protein acts as a pathogenicity determinant that leads to severe symptoms during the co-infection of BrYV and PEMV 2.

## 5. Conclusions

In this study, we demonstrated that the cell death induced by P0^BrA^ was accompanied by the accumulation of ROS and induction of *PR1* gene expression. We also showed that this cell death phenotype could be delayed by SA pretreatment. The comparison of the biological functions of the P0 proteins from the three BrYV genotypes demonstrated that transiently expressed P0^BrB^ or P0^BrC^ induced a significantly delayed and milder cell death response compared with P0^BrA^. However, they can also suppress local and systemic RNA silencing, such as P0^BrA^. Several residues in P0^BrA^ (Val70, Thr152, Pro163, Gln193, His227, and Phe228) essential for inducing cell death were identified by comparative analysis of the P0 proteins. Here, we also showed that all three BrYV genotypes had a synergistic interaction with PEMV 2 in *N. benthamiana*. This study provides theoretical guidance on controlling the viral disease caused by poleroviruses in the future.

## Data Availability

The data presented in this study are available within the article.

## Supplementary Materials

The following are available online at https://www.mdpi.com/article/10.3390/biology10111076/s1, Table S1: Sequences of primers used in this study.
