# Supplementary material for: Comparative Analysis of Biological Characteristics among P0 Proteins from Different Brassica Yellows Virus Genotypes
Source: Biology (Basel). 2021 Oct 21;10(11):1076. doi: 10.3390/biology10111076 (PMC8614689; doi:10.3390/biology10111076)
Supplement: Supplementary file 1 [file biology-10-01076-s001.zip › biology-1412377-supplementary.pdf]

Table S1. Sequences of primers used in this study.

Primer	Sequence [a] (5′-3′)	Note
BrBP0XhoF	**CTCGAG**ATGCAATTCATTGCTCAC	pGD-P0^{BrB}-3Flag construction
P0ApaR	TAT**GGGCCC**TAGCTCCCAAAACCCTTCG	
BrCP0XhoF	**CTCGAG**ATGCAATTTGTTGCTCAC	pGD-P0^{BrC}-3Flag construction
P0ApaR	TAT**GGGCCC**TAGCTCCCAAAACCCTTCG	
P0I56LF	TGATATTTTTCTTCGCTCTC	pGD-I56L-3Flag construction
P0I56LR	ATCTTGAATTCATTGTTGCG	
P0V70IF	ACCACATCCACGATGACGTT	pGD-V70I-3Flag construction
P0V70IR	CTCCGAGAAGGAGAGGAAGC	
P0T152IF	TCTATCTGGACTAGAGATGC	pGD-T152I-3Flag construction
P0T152IR	CAGAAATCGCTGGAAATGTT	
P0R159EF	TGAAGAACGCCTCTTTCCTG	pGD-R159E-3Flag construction
P0R159ER	GCATCTCTAGTCCAAGTAGA	
P0P163SF	CTCTTTTCTGGCTGTCGAGA	pGD-P163S-3Flag construction
P0P163SR	GCGTCTTTCAGCATCTCTAG	
P0V181LF	GCTCGCTAATCTTGGCGAGC	pGD-V181L-3Flag construction
P0V181LR	TCCACAAGCGTGTGAGACCC	
P0D191GF	TATGGTGGCTGGTGAGCAAT	pGD-D191G-3Flag construction
P0D191GR	ACGCGGAGAAGCTCGCCAAC	
P0Q193EF	GATGAGGAATTTCACAACTC	pGD-Q193E-3Flag construction
P0Q193ER	AGCCACCATAACGCGGAGAA	
P0S197PF	TTCACAACCCCCGTCTTCTG	pGD-S197P-3Flag construction
P0S197PR	ATTGCTCATCAGCCACCATA	
P0H227YF	ATCTGGATTATTTCGATTGC	pGD-H227Y-3Flag construction
P0H227/228YR	TGGCAATCCTCCAAAAAGAA	
P0F228LF	ATCTGGATCATCTCGATTGC	pGD-F228L-3Flag construction
P0H227/228YR	TGGCAATCCTCCAAAAAGAA	
18S-1	GCAAGACCGAAACTCAAAGG	RT-qPCR detection for *18S ribosomal RNA*

		gene
18S-2	TGTTCATATGTCAAGGGCTGG	
212F22	TTAGCAGCCGTCATGAAATCGT	RT-qPCR detection for *NbPR1*
341R23	GGCGTAGAACCTTTAACCTGGGA	
BrAP0ClaF	CC**ATCGAT**ATGCAATTTGTAGCTC	PVX.P0^{BrA} construction
BrAP0SalhisR	GC**GTCGAC**TTAATGATGGTGGTGGTGTAC AAACATTTC	
PEM2797F	ATGTCAATAACGACGAGCGC	RT-PCR detection for PEMV 2
PEM3202R	CCAATGCAATCGAGTAGGGT	

[a] The underlined letters represent the site of mutation. The bold font represents the restriction sites.
